# Removal of Cesium and Strontium Ions from Aqueous Solutions by Thermally Treated Natural Zeolite

**DOI:** 10.3390/ma16082965

**Published:** 2023-04-07

**Authors:** Marin Șenilă, Emilia Neag, Claudiu Tănăselia, Lacrimioara Șenilă

**Affiliations:** INCDO-INOE 2000, Research Institute for Analytical Instrumentation, 67 Donath Street, 400293 Cluj-Napoca, Romania

**Keywords:** clinoptilolite, radionuclides, volcanic tuff, porous materials, water treatment, chemisorption, ion-exchange

## Abstract

The radionuclides of cesium (Cs) and strontium (Sr) are dangerous products of nuclear fission that can be accidentally released into wastewater. In the present work, the capacity of thermally treated natural zeolite (NZ) from Macicasu (Romania) to remove Cs^+^ and Sr^2+^ ions from aqueous solutions in batch mode was investigated by contacting different zeolite quantities (0.5, 1, and 2 g) of 0.5–1.25 mm (NZ1) and 0.1–0.5 mm (NZ2) particle size fractions with 50 mL working solutions of Cs^+^ and Sr^2+^ (10, 50, and 100 mg L^−1^ initial concentrations) for 180 min. The concentration of Cs in the aqueous solutions was determined by inductively coupled plasma mass spectrometry (ICP-MS), whereas the Sr concentration was determined by inductively coupled plasma optical emission spectrometry (ICP-OES). The removal efficiency of Cs^+^ varied between 62.8 and 99.3%, whereas Sr^2+^ ranged between 51.3 and 94.5%, depending on the initial concentrations, the contact time, the amount, and particle size of the adsorbent material. The sorption of Cs^+^ and Sr^2+^ was analyzed using the nonlinear form of Langmuir and Freundlich isotherm models and pseudo-first-order (PFO) and pseudo-second-order (PSO) kinetic models. The results indicated that the sorption kinetics of Cs^+^ and Sr^2+^ on thermally treated natural zeolite was described by the PSO kinetic model. Chemisorption dominates the retention of both Cs^+^ and Sr^2+^ by strong coordinate bonds with an aluminosilicate zeolite skeleton.

## 1. Introduction

Considering the current trend of diminishing power plants based on fossil fuels because of high carbon dioxide emissions and the increasing energy demand, nuclear power plants are considered one of the best alternatives to produce cheap and clean energy [1,2,3]. Security issues represent the primary concern that is still limiting the development of this sector because of the dangerous radioactive wastes that nuclear power plants produce. Thus, the production of radioactive wastes could lead to significant ecological problems. Among the wastes generated in nuclear power plants, about 85% are liquid radioactive wastes [4]. The radionuclides of cesium (Cs) and strontium (Sr) are the most critical products of nuclear fission [5].

The Cs and Sr radionuclides have high chemical and biological radiotoxicity, damaging the human body [6], and thus, proper management of wastes from nuclear power plants is mandatory. The common approaches for radioactive Cs^+^ and Sr^2+^ removal from aqueous solutions include ion exchange, adsorption, precipitation, electrolysis, and redox methods [7]. Zeolites have particular characteristics that make these materials suitable candidates for the treatment of wastewater containing Cs^+^ and Sr^2+^. They possess a high cation-exchange capability, good adsorption capacity, and thermal and chemical stability [8,9,10,11,12,13], and exhibit ion-exchange and chemisorption properties [14]. Zeolites are complex, crystalline inorganic polymers, and their structure consists of three-dimensional frameworks of SiO_4_ and AlO_4_ tetrahedral. The aluminum ion fills the location in the center of the tetrahedron of four oxygen atoms, and the isomorphous replacement of Si^4+^ by Al^3+^ induces a negative charge in the lattice [15,16,17]. The exchangeable alkali or alkaline earth metal cations present within the zeolite structure, such as sodium, potassium, magnesium, and calcium, balance this net negative charge. These cations may be exchanged by other cations present in the contact solution [18,19,20]. Compared to synthetic zeolites and other adsorbing materials, clinoptilolite is widespread, cost-effective, and an appropriate adsorbent for large-scale wastewater applications [21].

Several studies were carried out for the removal of radioactive Cs and Sr [1,2,3,4,5,6,22]. At the same time, other sorption experiments were performed for non-radioactive Cs^+^ and Sr^2+^ removal to calculate the mass concentration that can be retained by the adsorbents [23,24]. The reported studies on the Cs^+^ and Sr^2+^ removal from liquid media by various adsorbent materials disclosed different results, probably due to their specific chemical compositions, structure, pore volumes, or cation-exchange capacity. Also, the water matrices from where cations are removed play an essential role in the removal efficiency. Kubota et al. [22] studied the removal of cesium, strontium, and iodine from water. The results revealed a successful reduction of ^134^Cs and ^85^Sr from river water by zeolite and bentonite, whereas ^85^Sr was scarcely removed from seawater. To the best of our knowledge, research aiming to solve the pollution problems of radioactive Cs^+^ and Sr^2+^ using zeolite-derived adsorbents is very scarce compared with studies that address the removal of other metal cations. Considering that a natural and cost-effective material is used, with high applicability, this represents a significant gap in this topic. Thus, the contribution to eliminating radio pollution is an original and essential research topic. This study aims to assess the sorption capacity of natural zeolite towards radionuclides after a minimum pretreatment, representing a novelty and following the zero-pollution and zero-carbon action plan [25].

The physicochemical properties and structure of zeolites influence their performance. Thus, new information related to the sorption behaviors of natural zeolites from different quarries is necessary. Therefore, this study aimed to (1) investigate the influence of process parameters, such as zeolite quantity, zeolite particle size, contact time, initial concentration, and competing ions over the removal of Cs^+^ and Sr^2+^ from contaminated aqueous solutions by thermally treated zeolites; (2) assess the contribution of ion-exchange and chemisorption processes to the total retention of Cs^+^ and Sr^2+^ by natural zeolites; (3) analyze the sorption kinetics and equilibrium data of Cs^+^ and Sr^2+^ removal onto two different grain sizes of natural zeolites.

## 2. Materials and Methods

### 2.1. Chemicals

Ultrapure water (18 MΩ cm^−1^) and analytical reagent-grade chemicals were used to prepare the solutions. The zeolite samples were digested using Emsure^®^ ACS premium grade acids HNO_3_ 65%, HCl 37%, and HF 40%, supplied from Merck (Darmstadt, Germany). The chemical analyses were performed according to the methodology described in previous work [12]. A single component Cs standard solution of 1000 mg L^−1^ purchased from Merck (Darmstadt, Germany) was used for external calibration of the inductively coupled plasma mass spectrometer, ICP-MS, ELAN DRCII (Perkin-Elmer, Woodbridge, ON, Canada). CRM BCS-CRM 375/1 soda feldspar (Bureau of Analyzed Samples, Middlesbrough, UK) was analyzed for quality control in total metal and oxides concentration with satisfactory accuracy. 

### 2.2. Zeolite Collection, Preparation, and Characterization

The zeolitic tuff was obtained from a quarry located in Macicasu, Cluj County, Romania. Particle size fractions of 0.5–1.25 mm (NZ1) and 0.1–0.5 mm (NZ2) were obtained by crushing and granulometric separation on sieves and then activated at 200 °C for 3 h. The aliquot parts from each granulometric fraction were ground in the laboratory using a micronization system (PilotMill-2 FPS1015, Como, Italy) to obtain a particle size of <0.05 mm for the analytical characterization (determination of major oxides, metals, exchangeable cations content). Total surface area, pore radius, and total pore volumes were measured on fractions of 0.5–1.25 mm and 0.1–0.5 mm particle sizes.

The content of major elements (Al, Ca, Mg, K, Na, Fe, Mn) in the zeolite samples were measured after microwave acid digestion of 1 g of zeolite sample with 14 mL mixture of HNO_3_ 65%: HCl 37%: HF 40% (3:9:2, *v:v:v*) using an Xpert system (Berghof, Eningen, Germany). A volume of 20 mL of saturated H_3_BO_3_ was added to neutralize the excess of HF. The resulting slurry was filtered through a 0.45 μm membrane filter and diluted with ultrapure water to a final volume of 100 mL. The resulting solutions were analyzed for metals using a dual viewing ICP-OES Optima 5300DV (Perkin Elmer, Norwalk, CT, USA), in radial viewing, after external calibration over the range 0–20 mg L^−1^ for each analyzed element. The concentrations of major oxides were calculated from the total elements concentration [12]. The SiO_2_ and loss of ignition (LOI) were measured by the gravimetric method [26], in triplicate. The cation-exchange capacity (CEC) [27] was estimated from the sum of major cations (K^+^, Na^+^, Ca^2+^, and Mg^2+^) concentrations extracted in 1 M ammonium acetate solution and measured by ICP-OES in radial viewing mode. Zeolitic tuff morphology (total surface area, total pore volume, and average pore radius) was evaluated according to the methodologies described in our previous work [12]. 

### 2.3. Batch Sorption Experiments

Stock solutions of Cs^+^ (1000 mg L^−1^) and Sr^2+^ (1000 mg L^−1^) were prepared by dissolving 1.266 g CsCl and 1.809 g SrCl_2_, respectively, in 1000 mL of ultrapure water. The salts were of analytical purity and purchased from Merck, Darmstadt, Germany. The working solutions with initial Cs^+^ and Sr^2+^ concentrations of 10, 50, and 100 mg L^−1^ were prepared by appropriate dilution of the concentrated stock solutions with ultrapure water. The experiments were performed in batch mode, contacting different quantities of NZ1 and NZ2 (0.5, 1, and 2 g) with 50 mL working solutions of Cs^+^ and Sr^2+^ at a stirring rate of 200 rpm at room temperature (22 ± 2 °C). Samples were taken at different contact times (1, 3, 5, 7, 10, 30, 60, 90, 120, and 180 min) and further filtered using 0.45 μm polyethersulfone (PES) syringe filters (Whatman, Clifton, NJ, USA). The concentration of Sr was determined using ICP-OES, whereas Cs concentration, due to its low analytical signal in ICP-OES, was determined using ICP-MS. All the experiments were performed in triplicate, and the average value was used.

The Cs^+^ and Sr^2+^ amount in the adsorbent phase, *q_e_* (mg/g) was calculated using Equation (1), whereas removal efficiency, *E* (%) was calculated using Equation (2):

(1) $$q_{e}=\frac{\left(C_{o}{\;-\;C}_{e}\right)}{m}\cdot \frac{V}{1000}$$

(2) $$E(\%)=\frac{{(C}_{o}{\;-\;C}_{e})}{C_{o}}\cdot 100$$

where *q_e_* is the Cs^+^ and Sr^2+^ amount adsorbed per gram of adsorbent at equilibrium (mg g^−1^), *V* is the volume of solution (mL), *m* is the weight of natural zeolite (g), *C_e_* is the equilibrium Cs^+^ and Sr^2+^ concentrations (mg L^−1^), and *C_0_* is the initial Cs^+^ and Sr^2+^ concentrations (mg L^−1^) [28].

In order to appraise the possible release of Cs^+^ and Sr^2+^ from natural zeolite before its use in the batch experiments, and to account for these concentrations in the calculation of the removal efficiency, 0.5, 1, and 2 g, of the NZ1 and NZ2 were mixed with 50 mL of ultrapure water at a stirring rate of 200 rpm for 2 h at room temperature, then the resulting solutions were filtered by 0.45 μm PES syringe filter (Whatman, Clifton, NJ, USA). To evaluate if the retention of Cs^+^ and Sr^2+^ on zeolites is based on ion exchange or chemisorption, the zeolite that previously retained Cs^+^ and Sr^2+^ when contacted with 50 mL of solution with a concentration of 100 mg L^−1^ for 180 min was subjected to desorption. For this, zeolite was mixed with ammonium acetate solution 1 M at a ratio solid: liquid of 1:50, at a stirring rate of 200 rpm for 3 h at room temperature, and finally the resulting solutions were filtered by a 0.45 μm PES filter. The quantities of Cs and Sr desorbed from the solid phase were determined considering the initial amount of zeolite and the final volume of extraction solutions [12,29].

In order to evaluate the influence of the zeolite structure on the sorption mechanisms, the batch experimental data for Cs^+^ and Sr^2+^ removal were correlated with the XRF and FTIR results performed on zeolite tuff before and after the sorption of cations.

The zeolite samples containing the sorbed cations were separated from the aqueous solution by filtration and then dried for 2 h at 105 °C. The infrared spectra of the zeolite samples were recorded on 1% KBr pellets in the 4000–400 cm^−1^ range, with a spectral resolution of 2 cm^−1^, using a BX II Fourier transform infrared spectrophotometer (Perkin Elmer, Waltham, MA, USA). XRF analyses were performed using a Bruker Tracer 5i portable X-ray fluorescence with a 5 kV and 4-watt X-ray source, 8 μm Be window, and 8 mm spot collimator.

### 2.4. Equilibrium and Kinetics Studies

The sorption experiments and kinetics studies were carried out in batch mode by adding 0.5 g of NZ1 and NZ2 to 50 mL of various Cs^+^ and Sr^2+^ solutions of different initial concentrations for 180 min. 

The isotherm parameters for Cs^+^ and Sr^2+^ sorption by thermally treated natural zeolite were estimated using nonlinear forms of Langmuir and Freundlich isotherm models. The equations of the used models were presented in our previous paper [12]. The nonlinear forms of pseudo-first (PFO) and pseudo-second-order (PSO) kinetic models were used to investigate the sorption kinetics of Cs^+^ and Sr^2+^ on NZ1 and NZ2. The nonlinear regression analysis of Cs^+^ and Sr^2+^ sorption on thermally treated natural zeolite was performed using the OriginPro software, version 2020b, OriginLab Corporation, Northampton, MA, USA.

## 3. Results and Discussion

### 3.1. Characteristics of Thermally Treated Volcanic Tuffs

The major oxides content in the zeolites is presented in Table 1. The Si/Al ratio > 4 and Na + K > Ca in zeolitic samples indicate the presence of clinoptilolite as a major constituent [30].

Generally, the concentration of major oxides (Table 1) was not affected by the grain sizes of the zeolite samples. Moreover, their concentration was not significantly different than that measured in the zeolitic tuff from the same quarry studied in previous work [31], except for K_2_O, which was 30% higher in the current work. Also, X-ray diffraction (XRD) analysis showed clinoptilolite as the main zeolite mineral and quartz, muscovite, and albite as minor phases in the zeolitic tuff from this quarry [31]. Moreover, SEM–EDX studies revealed that the zeolite sample contains mainly clinoptilolite [31]. 

The crystalline structure of clinoptilolite is stable at high temperatures. This was confirmed by thermogravimetric analysis (TGA) conducted at a temperature of up to 1000 °C. According to the TGA analysis, the samples showed high thermal stability [30], which is characteristic of clinoptilolite [32]. 

The total surface area is 46.1 m^2^ g^−1^ in NZ1 and 43.8 m^2^ g^−1^ in NZ2, whereas the total pore volume is 0.093 cm^3^ g^−1^ in NZ1, and 0.082 cm^3^ g^−1^ in NZ2, respectively. The average pore radius is 36 Å in NZ1 and 36 Å in NZ2, less than 50 nm which classifies the samples as mesoporous materials [32]. No important differences between larger (0.5–1.25 mm) and smaller grain size (0.1–0.5 mm) samples were observed since the main contribution to the total surface area is given by the internal specific surface area of zeolite which is not meaningly modified by grinding. The surface area and total pore volume are important physical parameters in the sorption process. Concentrations of exchangeable cations are presented in Table 2.

The main contribution of the exchangeable major cations to the total CEC decreased in the order Ca^2+^ > K^+^ >> Na^+^ ≅ Mg^2+^. The theoretic CEC value calculated as the total content of K, Na, Ca, and Mg in NZ1 is 239.7 meq/100 g, which consists of 30.6 meq K^+^/100 g, 43.5 meq Na^+^/100 g, 134.6 meq Ca^2+^/100 g, and 31.0 meq Mg^2+^/100 g, whereas in NZ2 the theoretical CEC value is 239.4 meq/100 g, containing 30.1 meq K^+^/100 g, 39.0 meq Na^+^/100 g, 140.7 meq Ca^2+^/100 g, and 29.5 meq Mg^2+^/100 g. These results show that K^+^ is the most mobile cation. On average, 78% of K, 46% of Ca, 7% of Na, and 11% of Mg cations from the studied zeolitic materials are in an exchangeable form and can be involved in ion-exchange processes.

### 3.2. Release of Cs^+^ and Sr^2+^ from Natural Zeolites to the Aqueous Solution

In order to evaluate the possible release of Cs^+^ and Sr^2+^ from natural zeolite into the aqueous solution during the batch experiments, different NZ1 and NZ2 quantities (0.5, 1, and 2 g) were mixed with 50 mL of ultrapure water for 180 min. The concentration of released Cs^+^ was in all cases below LOQ (0.5 µg L^−1^). In the case of Sr^2+^, the measured released concentrations varied between 3.1 µg L^−1^ (<LOQ) and 1.02 µg L^−1^ (maximum concentration released from zeolite NZ2).

The concentrations of Sr^2+^ measured in these experiments were subtracted from the equilibrium concentrations of Sr^2+^ measured in the batch experiments at the corresponding contact time and zeolite grain size.

### 3.3. Removal Efficiency (E%) from Contaminated Solutions

**Figure 1:**
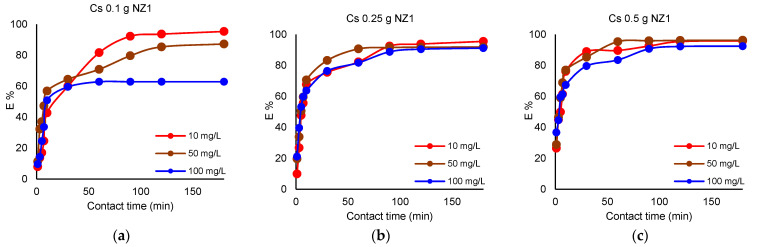
Removal efficiency (%) of Cs^+^ by different NZ1 quantities; (**a**) 0.1 g, (**b**) 0.25 g, (**c**) 0.5 g; V = 50 mL; 200 rpm; 22 ± 2 °C, 180 min.

**Figure 2:**
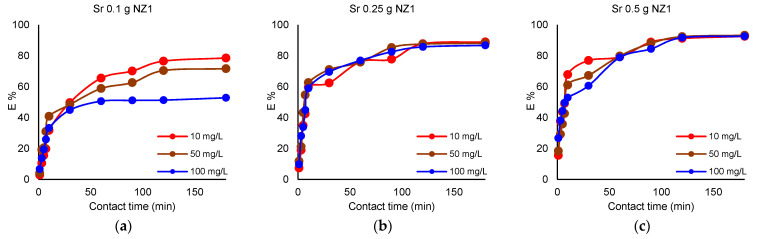
Removal efficiency (%) of Sr^2+^ by different NZ1 quantities; (**a**) 0.1 g, (**b**) 0.25 g, (**c**) 0.5 g; V = 50 mL; 200 rpm; 22 ± 2 °C, 180 min.

The experiments were carried out using different NZ1 and NZ2 dosages (0.5, 1, 2 g) which were contacted with 50 mL of Cs^+^ and Sr^2+^ solution (concentrations of 10, 50, 100 mg L^−1^) for 180 min. The removal of Cs^+^ and Sr^2+^ by different NZ1 quantities is presented in Figure 1 and Figure 2.

**Figure 3:**
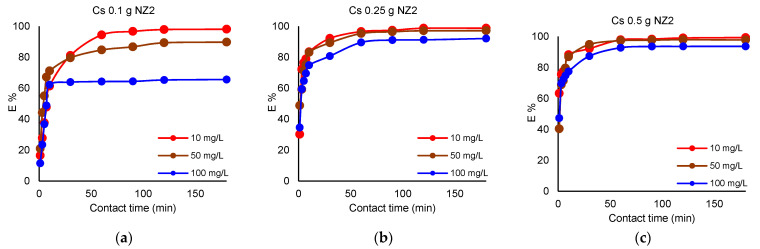
Removal efficiency (%) of Cs^+^ by different NZ2 quantities; (**a**) 0.1 g, (**b**) 0.25 g, (**c**) 0.5 g; V = 50 mL; 200 rpm; 22 ± 2 °C, 180 min.

**Figure 4:**
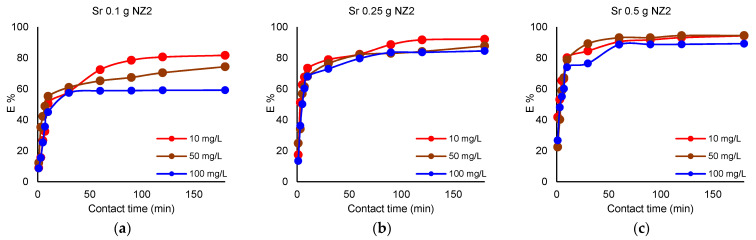
Removal efficiency (%) of Sr^2+^ by different NZ2 quantities; (**a**) 0.1 g, (**b**) 0.25 g, (**c**) 0.5 g; V = 50 mL; 200 rpm; 22 ± 2 °C, 180 min.

The removal of Cs^+^ and Sr^2+^ by different NZ2 quantities is presented in Figure 3 and Figure 4.

#### 3.3.1. Influence of Zeolite Quantities on Removal Efficiency 

The influence of the zeolite dosage on the Cs^+^ and Sr^2+^ removal efficiency is mainly observed when a smaller amount (0.1 g) of adsorbent was used. Thus, when 0.1 g NZ1 is used as an adsorbent for Cs^+^, the maximum removal efficiency (E%) is 63.6% at an initial concentration of 100 mg L^−1^, 87.2% at an initial concentration of 50 mg L^−1^, and reaches 95.3% when the initial concentration is only 10 mg L^−1^. Increasing NZ1 dosage to 0.25 g leads to a maximum E% for Cs^+^ of 92%, regardless of the initial concentrations of the solution. A higher dosage of 0.5 g NZ1 increased the maximum E% for Cs^+^ to 96% when the initial concentrations of the solution were 10 mg L^−1^ and 50 mg L^−1^ and 93% when the initial concentration was 100 mg L^−1^. In the case of Sr^2+^, when 0.1 g NZ1 is used, the maximum (E%) is 51.3% when the initial concentration of the solution is 100 mg L^−1^, 71.6% when the initial concentration of the solution is 50 mg L^−1^ and 79.5% when the initial concentration is 10 mg L^−1^. The 0.25 g NZ1 dosage results in a maximum E% of 88%, whereas the dosage of 0.5 g of NZ1 conducts an increasing removal efficiency of 93%.

As presented in Figure 3 and Figure 4, if 0.1 g of NZ2 is used for Cs^+^ removal, a maximum E% of 65.6% is obtained at a concentration of 100 mg L^−1^, 89.8% maximum E% if the initial concentration of the solution is 50 mg L^−1^, and 98.1% maximum E% at the initial concentration of 10 mg L^−1^. If 0.25 g NZ2 is used, E% for Cs^+^ reach about 92% at the concentration of 100 mg L^−1^, and about 97–98% at lower concentrations of 50 mg L^−1^ and 10 mg L^−1^, respectively. The dosage of 0.5 g NZ2 removed over 99% Cs^+^ from the solution with an initial concentration of 10 mg L^−1^. In the case of Sr^2+^, when 0.1 g NZ2 is used, the maximum E% is 59.1% at the initial concentration of 100 mg L^−1^, 74.3% if the initial concentration of the solution is 50 mg L^−1^, and 81.7% when the initial concentration is 10 mg L^−1^. The 0.25 g NZ2 dosage results in a maximum removal efficiency of about 92%, whereas the dosage of 0.5 g NZ1 increases removal efficiency to over 94%. Results demonstrated that adsorbed Cs^+^ and Sr^2+^ ions gradually increased with the adsorbent dosage, both for NZ1 and NZ2.

As the concentration of Cs^+^ and Sr^2+^ increases in the solution, the driving force increases to overcome the mass transfer resistance of strontium from the aqueous solution into zeolite, which increases the sorption capacity of the zeolite.

#### 3.3.2. Influence of Contact Time

The removal efficiency of Cs^+^ increased within 60 min of removal when the solutions were contacted with 0.1 g of zeolites reaching the maximum at this contact time. By increasing the zeolite amount, the removal efficiency of Cs^+^ rapidly increased and reached equilibrium after 30 min. The removal efficiency of Sr^2+^ slowly increased as compared with Cs^+^ removal. When the solutions were contacted with 0.1 g of zeolites, the maximum removal efficiency was achieved after 120 min. Furthermore, the removal efficiency increased with the increase of the zeolite quantity. Li et al. [6] reported a maximum removal efficiency for Sr^2+^ at 30 min.

### 3.4. Influence of Zeolite Grain Sizes on Removal Efficiency (E%)

The effect of particle size of NZ1 and NZ2 is presented in Figure 1, Figure 2, Figure 3 and Figure 4. Cs^+^ and Sr^2+^ removal efficiencies are strongly influenced by zeolite particle size. So, the samples with smaller particle sizes were more efficient for cations sorption than the samples with larger particle sizes due to their larger external surface area available for the sorption. The particle size reduction increases the external surface area available for the interaction with ions in the solution and leads to a shorter diffusion path length. The diffusion of cations from the external to interparticle sites in zeolites is slow because of their interaction with the surface functional group [33]. This finding was also reported in previous studies [12,34], which showed that smaller particle sizes have an increased number of active sites.

### 3.5. Effect of Interfering Ions

Sorption properties and zeolite selectivity are strongly influenced by exchangeable cation properties, solution concentration, presence of other cations, and zeolite characteristics. Zeolites with a high Si/Al ratio have a higher affinity for cations with lower charge densities, such as Cs, while zeolites with a lower Si/Al ratio favor the retention of cations with higher charge densities [24]. Usually, the zeolite used in our experiments with a high Si/Al (>4), should prefer Cs^+^ and Sr^2+^ to cations such as Na^+^, K^+^, Ca^2+^, and Mg^2+^. To evaluate the preference of natural zeolite used in this study for the Cs^+^ and Sr^2+^ sorption in the presence of competitor cations, a 50 mL multicomponent solution containing 100 mg L^−1^ of Na^+^, K^+^, Ca^2+^, Mg^2+^, Cs^+^, and Sr^2+^ was contacted for 60 min with 0.1 g of NZ1 in batch mode at a stirring rate of 200 rpm at room temperature (22 ± 2 °C). After filtration, Cs^+^ and Sr^2+^ concentrations in the solution were measured. Compared with the removal from single component solutions, a decrease to 73.5% from the maximum sorption capacity for Cs^+^ and 64.7% from the maximum sorption capacity of Sr^2+^ was recorded.

### 3.6. Retention Mechanisms

Ion exchange and chemisorption are the two main mechanisms that contribute to the retaining of cations by zeolites [15]. Ion exchange involves the replacement of the exchangeable cations (Na^+^, K^+^, Ca^2+^, and Mg^2+^) from the zeolite crystalline framework with other cations present in the contact solution. The cations retained in zeolite by ion exchange are weakly bounded and can be replaced by NH_4_^+^. In contrast, if the cations are retained by stronger chemical bonds (chemisorption) cannot be replaced by NH_4_^+^ [15,29]. The zeolites previously maintained in a 50 mL solution of 100 mg L^−1^ Cs^+^ and Sr^2+^ were contacted with a 1 M ammonium acetate solution (ratio 1:50) for desorption to assess the Cs^+^ and Sr^2+^ retained due to the ion exchange process. The results revealed that 44.4% of Cs^+^ and 38.2% of Sr^2+^ were desorbed from zeolite being replaced by NH_4_^+^, which means that these cations are retained mainly by chemisorption in this type of zeolite. In addition, the data were analyzed using the Dubinin–Radushkevich (D–R) isotherm model to investigate whether the sorption process of Cs^+^ and Sr^2+^ was physical or chemical. The mean free energy (E) values calculated from the D-R isotherm model for Cs^+^ sorption on NZ1 and NZ2 were 9.2 kJ mol^−1^ and 12.1 kJ mol^−1^, respectively. Regarding Sr^2+^ sorption on NZ1 and NZ2, the E values were 8.0 kJ mol^−1^ and 9.0 kJ mol^−1^, respectively. Thus, the process of Cs^+^ and Sr^2+^ sorption on both zeolites occurred by chemisorption.

**Figure 5:**
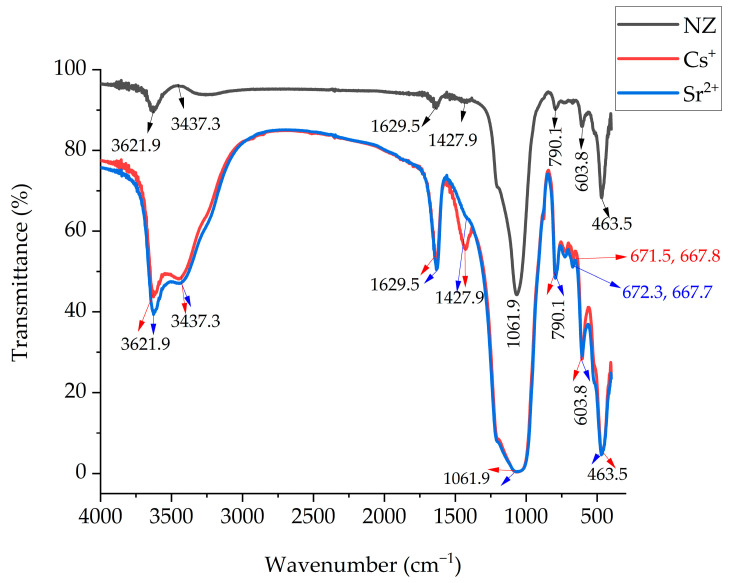
FTIR spectra of the zeolitic tuff sample before and after Cs^+^ and Sr^2+^ sorption.

The ICP results have been linked with IR spectroscopic studies to assess the retention mechanisms. FTIR spectra of the zeolitic tuff sample before and after Cs^+^ and Sr^2+^ sorption were recorded in the range of 400–4000 cm^−1^ to identify the presence of functional groups responsible for the sorption process (Figure 5). There are several absorption peaks, indicating the complex nature of the zeolite. The peaks around 1630, and 3622 cm^−1^ were attributed to H–O–H bending O–H bonding (hydroxyl groups) vibrations and water absorption bands of the clinoptilolite, respectively [35,36]. The main characteristic bands of clinoptilolite were found at the wavelengths of 464 cm^−1^ resulting from the stretching vibrations of the Al–O bonds, at 604 cm^−1^ which is assigned to the vibration of the external linkage of the tetrahedral, at 790 and 1063 cm^−1^ (the most intense band characteristic to silicates) which are ascribed to Si–O–Si and Al–O–Si bonds due to asymmetric stretching vibrations [15,36].

The most important changes in IR spectra following the Cs^+^ and Sr^2+^ sorption are observed in the range of the pseudo-lattice vibrations, that is, 700–600 cm^−1^, as it has been previously reported for other heavy metals sorption [29]. Thus, the bands in the range of 700–655 cm^−1^ attributed to the vibrations of rings in the zeolite structure have been investigated in more detail. In this range, the band at 672 cm^−1^ is observed when Cs^+^ is sorbed, whereas the band at 668 cm^−1^ appears when Sr^2+^ is sorbed. Consequently, these two bands are characteristic of sorption through symmetrical stretching vibrations of the Si–O bond existing in the rings of the zeolite. 

The change in the intensity of these bands can give information related to the amount of Cs^+^ and Sr^2+^ sorbed in the zeolite structure. Even if an increase in the intensity of the two bands was observed with an increase of Cs^+^ and Sr^2+^ initial concentration, a more precise quantification of the amount of Cs^+^ and Sr^2+^ sorbed on zeolite can be obtained after ICP measurement of the two cations remained in the solution.

As estimated from the adsorption and desorption of the two cations with 1 M ammonium acetate solution, chemisorption dominates the retention for the monovalent Cs^+^ and bivalent cation of Sr^2+^, and this can be due to a lesser extent by the zeolite structure.

A possible explanation of the higher contribution of chemisorption to the retention of Cs^+^ and Sr^2+^ is that the stable inner-sphere complexes are formed at the zeolite surface [37]. The higher ionic radius of Cs^+^ and Sr^2+^ compared with the major cations from zeolite may prevent the diffusion of these ions in the zeolite framework to participate in the ion exchange process. Thus, the two cations form mainly strong coordinate bonds with an aluminosilicate sorbent skeleton.

**Figure 6:**
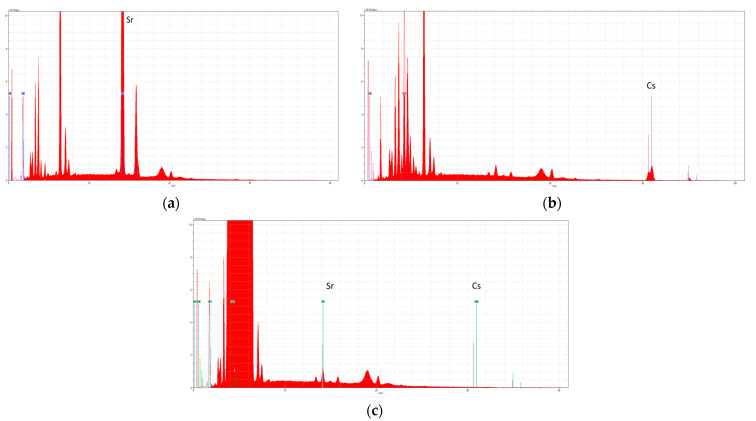
XRF spectra of zeolite after the sorption of Sr^2+^ (**a**), after the sorption of Cs^+^ (**b**), and before the sorption experiments (**c**).

The spectra of zeolite before and after the sorption of Cs^+^ and Sr^2+^ were registered by XRF (Figure 6). There was no Cs^+^ in the zeolite before sorption, but its signal was detected after sorption. In the case of Sr^2+^, a signal was observed even before extraction, due to a concentration of about 200 mg kg^−1^ in the natural zeolite, which significantly increased after the extraction.

### 3.7. Adsorption Isotherms and Kinetics

**Figure 7:**
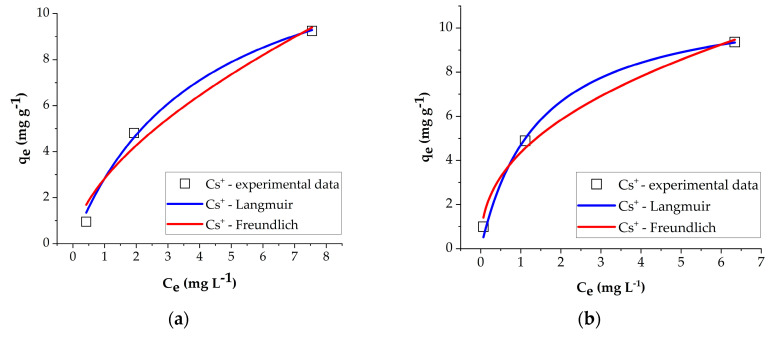

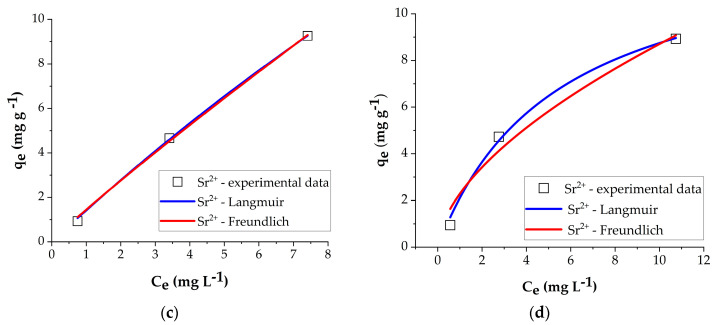
Nonlinear fitting of Langmuir and Freundlich isotherm models for the Cs^+^ sorption on NZ1 (**a**), Cs^+^ sorption on NZ2 (**b**), Sr^2+^ sorption on NZ1 (**c**), and Sr^2+^ sorption on NZ2 (**d**).

The nonlinear fitting of Langmuir and Freundlich isotherm models for the Cs^+^ and Sr^2+^ sorption on NZ1 and NZ2 at different initial concentrations is presented in Figure 7.

The Langmuir isotherm parameters, *q_max_* (the maximum sorption capacity, mg g^−1^) and *K_L_* (Langmuir constant, L mg^−1^), and the Freundlich isotherm parameters, *K_F_* (related to sorption capacity, mg^1−1/*n*^ L^1/*n*^ g^−1^) and 1/*n* (the sorption intensity) [12] are listed in Table 3. Table 3 shows that the experimental data for Cs^+^ and Sr^2+^ sorption on NZ1 and NZ2 were slightly better fitted using the Langmuir isotherm model than with the Freundlich isotherm model. For instance, the values of R^2^ determined using the Langmuir isotherm model for Cs^+^ sorption on NZ1 and NZ2 were 0.9945 and 0.9932, respectively. These values were slightly higher than that determined using the Freundlich isotherm model (R^2^ = 0.9722 and R^2^ = 0.9916, respectively). A similar trend was observed for Sr^2+^ sorption by both NZ1 and NZ2. This indicates that the zeolite surface is homogeneous, and monolayer sorption of Cs^+^ and Sr^2+^ on a thermally treated zeolite surface can take place [5]. The obtained results are in accordance with previous studies [38,39]. Mimura and Akiba [8] investigated the removal of Cs^+^ and Sr^2+^ on synthetic zeolite P and found that the sorption of the studied ions obeyed the Langmuir isotherm model. Also, Lee et al. [39] found that the Langmuir isotherm model fitted better than the Freundlich and Dubinin–Radushkevich isotherms models, the sorption of Cs^+^ and Sr^2+^ on zeolitic materials synthesized from Jeju volcanic rocks.

According to the data presented in Table 3, the maximum sorption capacity for Cs^+^ was 14.22 mg g^−1^ on NZ1 and 11.46 mg g^−1^ on NZ2, whereas the maximum sorption capacity for Sr^2+^ was 68.88 mg g^−1^ on NZ1 and 13.46 mg g^−1^ on NZ2.

In a study on the removal of Sr^2+^ from an aqueous solution using synthetic zeolite, Arraisi et al. [23] obtained a maximum adsorption capacity of 2.36 mmol g^−1^, which decreased to 1.60 mmol g^−1^ when a binary solution with Ba^2+^ was used. Bakusi et al. [40] evaluated the adsorption of Sr^2+^ from an aqueous media by TiO2-modified zeolite and reported an adsorption capacity of 9.632 mg g^−1^. Belviso et al. [41] used zeolites synthesized from volcanic ash for Cs removal and reported maximum adsorption capacities in the range of 31.75–63.69 mg g^−1^. Borai et al. [42] used zeolites impregnated with calix [4]arene for Cs removal and obtained maximum adsorption capacities ranging between 1.201 and 1.865 mmol g^−1^. Lee et al. [43] prepared geopolymers containing nano-crystalline zeolites and reported a maximum adsorption capacity for Cs^+^ of 15.24 mg g^−1^. Abdel Moamen et al. [44] reported maximum adsorption capacities of 15.32–19.29 mEq g^−1^ for Sr^2+^ and 5.74–6.72 mEq g^−1^ for Cs^+^ on synthetic nano-sized zeolite using the nonlinearized form of Langmuir isotherm model. Synthesized zeolite Y had a maximum adsorption capacity of 2.401 mmol g^−1^ for Cs+ and 1.527 mmol g^−1^ for Sr^2+^ [45]. Adsorption studies on a modified zeolite with nano iron particles for strontium removal found adsorption capacities in the range of 9.08–32.83 mg g^−1^ from solution with initial concentrations of Sr^2+^ ranging between 50 and 200 mg L^−1^ [46]. A maximum adsorption capacity of 84.12 mg g^−1^ for Sr^2+^ removal on modified zeolite with nano iron particles was obtained [46]. Lee et al. [39] reported maximum adsorption capacities of 154.84 mg/g for Sr^2+^ and 144.01 mg g^−1^ for Cs^+^ removal by zeolitic materials. Higher adsorption capacities ranging between 54.5 and 45.2 mg g^−1^ for Cs^+^ and between 30 and 21 mg g^−1^ for Sr^2+^ by clinoptilolite-rich zeolites (with smaller particle sizes) were reported by Sterba et al. [47]. Our study demonstrated that the natural zeolite from the studied quarry is highly effective for Cs^+^ and Sr^2+^ removal after a minimum pretreatment. Both particle fractions, 0.5–1.25 mm (NZ1) and 0.1–0.5 mm (NZ2), can be easily prepared by crushing and granulometric separation on sieves in a factory and then activated at a temperature of only 200 °C to eliminate water from zeolite pores. The cost of the zeolite used in this study was 0.2 USD kg^−1^, much less than that of synthetic zeolites, (approximately 50 US dollars kg^−1^).

The kinetic parameters, *q_t_* (the amount adsorbed at time *t*, mg g^−1^), *k*_1_ (the first-order rate constant, min^−1^), and *k*_2_ (the second-order rate constant, g mg·min^−1^) obtained by applying the nonlinearized form of the PFO and PSO for Cs^+^ and Sr^2+^ sorption on NZ1 and NZ2 are presented in Table 4.

The calculated *q_e_* (*q_e_, _calc_*) values estimated by the PFO are not close to the experimental *q_e_* values (*q_e_, _exp_*), indicating that the model is not suitable to describe the Cs^+^ and Sr^2+^ sorption kinetics on NZ1 and NZ2. However, the *q_e_, _calc_* from PSO are close to the *q_e_, _exp_*, and the R^2^ values are higher than those obtained for the PFO. Thereby, the Cs^+^ and Sr^2+^ sorption kinetics on NZ1 and NZ2 followed PSO rather than PFO. This indicates that the sorption process of Cs^+^ on NZ1 and Sr^2+^ on NZ2 occurs as chemisorption.

El-Rahman et al. [48] studied the kinetics of Cs^+^ and Sr^2+^ on synthetic zeolite A using linear forms of PFO and PSO models and found that PFO was not adequate to predict the sorption kinetics of both ions. Also, similar results were found for the sorption of Sr^2+^ and Cs^+^ on zeolitic materials synthesized from Jeju volcanic rocks [39] and natural zeolites [5]. Moreover, it can be observed that the *k*_2_ constants obtained for Cs^+^ sorption are higher than those obtained for the sorption of Sr^2+^ on both zeolites. This agrees with the findings of Kwon et al. [1] and is ascribed to the strong hydration of Sr^2+^ [1].

The two cations are sorbed mainly by strong coordinate bonds with an aluminosilicate zeolite skeleton. Thus, they are difficult to extract back. However, the production of secondary solid waste is a key challenge in the sorption process. To be deposited after sorption without the risk of release to the environment, Abdollahi et al. [5] developed a stabilization procedure by heating the resulting secondary solid waste at temperatures ranging between 700 and 1100 °C for 2, 6, and 12 h. The leaching tests showed that Cs^+^ and Sr^2+^ were well stabilized in the natural zeolite structure, and this represents a practical solution to manage the zeolite that retained these cations.

Considering that natural zeolite is a widespread and cheap material with good adsorption capacity, it represents a promising alternative for the treatment of water contaminated with radioactive Cs^+^ and Sr^2+^ after a minimum pretreatment procedure. Therefore, it can be used for the decontamination of large amounts of contaminated water.

## 4. Conclusions

In this study, the use of thermally treated natural zeolite from a quarry from NW of Romania showed a high potential for the removal of Cs^+^ and Sr^2+^ from aqueous solutions. Two different particle sizes of (0.5–1.25 and 0.1–0.5 mm) thermally treated zeolite at 200 °C were characterized and tested in batch experiments. The experiments were carried out using different NZ1 and NZ2 dosages (0.5, 1, 2 g) which were contacted with 50 mL of Cs^+^ and Sr^2+^ solution (10, 50, 100 mg L^−1^ initial concentrations) for 180 min. A good sorption capacity is due to the high cation-exchange capacity, surface area, and porosity. The removal efficiency is influenced by zeolite dosage, initial concentration, contact time, and grain sizes. Maximum removal efficiency of 99.3% for Cs^+^ when a dosage of 0.5 g zeolite with a grain size of 0.1–0.5 mm was contacted for 180 min with a solution with an initial concentration of 10 mg L^−1^. For Sr^2+^ removal from solution, a maximum removal efficiency of 92.1% when a dosage of 0.5 g zeolite with a grain size of 0.1–0.5 mm was contacted for 180 min with a solution with an initial concentration of 10 mg L^−1^. The competing ions of Na^+^, K^+^, Ca^2+^, and Mg^2+^ from a multi-component solution with concentrations of 100 mg L^−1^ each decreased the maximum adsorption capacities to 73.5% for Cs^+^ and 64.7% for Sr^2+^. The sorption mechanism was investigated based on the adsorption and desorption studies correlated with FTIR spectroscopy, and it was found that the chemisorption dominates the retention of both Cs^+^ and Sr^2+^. Thus, mainly strong coordinate bonds with an aluminosilicate sorbent skeleton are formed. At the same time, some cations are retained by ion exchange and can be replaced from the zeolite structure by other cations. Therefore, the natural zeolite from Macicasu (Romania) is efficient and has a high adsorption capacity for Cs^+^ and Sr^2+^ from aqueous solutions. Since zeolite is a natural and inexpensive material, it should be considered a viable solution for the treatment of wastewater contaminated with Cs^+^ and Sr^2+^.

## Figures and Tables

**Table 1:** Chemical composition of zeolitic tuff from Macicasu quarry (average value ± stdev. (*n* = 3 parallel determinations).

Parameter	NZ1	NZ2
pH	8.31 ± 0.25	8.40 ± 0.28
SiO_2_ (%)	65.8 ± 2.2	66.1 ± 2.3
Al_2_O_3_ (%)	12.3 ± 0.8	12.5 ± 0.7
CaO (%)	3.77 ± 0.07	3.94 ± 0.08
MgO (%)	0.63 ± 0.05	0.60 ± 0.04
K_2_O (%)	1.44 ± 0.13	1.42 ± 0.11
Na_2_O (%)	1.35 ± 0.10	1.21 ± 0.12
Fe_2_O_3_ (%)	2.07 ± 0.12	2.05 ± 0.10
MnO (%)	0.03 ± 0.004	0.03 ± 0.004
LOI (%)	2.33 ± 0.65	2.21 ± 0.83
Others (%)	1.97	1.54

**Table 2:** Concentrations of exchangeable cations (CEC) of the NZ1 and NZ2 thermally treated samples (*n* = 3 parallel determinations).

Zeolite	Na^+^	K^+^	Ca^2+^	Mg^2+^	CEC
	mEq 100 g^−1^				
NZ1	3.2	24.5	61.7	3.3	92.6
NZ2	2.6	22.7	64.8	3.6	93.8

**Table 3:** Langmuir and Freundlich parameters for Cs^+^ and Sr^2+^ sorption on NZ1 and NZ2 at different initial concentrations.

Cation	Zeolite	Langmuir Isotherm			Freundlich Isotherm		
		q_max_	K_L_	R^2^	K_F_	*n*	R^2^
		(mg g^−1^)	(L mg^−1^)		(mg ^1−1/*n*^ L^1/*n*^ g^−1^)		
Cs^+^	NZ1	14.22	0.25	0.9945	2.83	0.59	0.9722
Cs^+^	NZ2	11.46	0.70	0.9932	4.36	0.42	0.9916
Sr^2+^	NZ1	68.88	0.02	0.9993	1.45	0.92	0.9985
Sr^2+^	NZ2	13.46	0.19	0.9956	2.29	0.58	0.9726

**Table 4:** Kinetic parameters for Cs^+^ and Sr^2+^ sorption on NZ1 and NZ2 at different initial concentrations.

Cation	Zeolite	Model	Parameters	10 mg/L	50 mg/L	100 mg/L
Cs^+^	NZ1	PFO	*k*_1_ (1 min^−1^)	0.18	0.21	0.23
			*q_e, calc_* (mg g^−1^)	0.92	4.65	8.59
			R^2^	0.9544	0.9498	0.81558
		PSO	*k*_2_ (g mg·min^−1^)	0.28	0.07	0.04
			*q_e, calc_* (mg g^−1^)	0.97	4.9	9.02
			R^2^	0.9795	0.9897	0.9326
			*q_e, exp_*	0.96	4.81	9.25
	NZ2	PFO	*k*_1_ (1 min^−1^)	1.01	0.38	0.57
			*q_e, calc_* (mg g^−1^)	0.91	4.72	8.70
			R^2^	0.510	0.890	0.73
		PSO	*k*_2_ (g mg·min^−1^)	1.47	0.13	0.10
			*q_e, calc_* (mg g^−1^)	0.96	4.95	9.21
			R^2^	0.854	0.989	0.948
			*q_e, exp_*	0.99	4.89	9.34
Sr^2+^	NZ1	PFO	*k*_1_ (1 min^−1^)	0.15	0.11	0.15
			*q_e, calc_* (mg g^−1^)	0.86	4.27	8.14
			R^2^	0.9532	0.9257	0.7846
		PSO	*k*_2_ (g mg·min^−1^)	0.21	0.03	0.02
			*q_e, calc_* (mg g^−1^)	0.93	4.69	8.79
			R^2^	0.9786	0.9676	0.8926
			*q_e, exp_*	0.93	4.66	9.26
	NZ2	PFO	*k*_1_ (1 min^−1^)	0.30	0.20	0.22
			*q_e, calc_* (mg g^−1^)	0.89	4.64	8.55
			R^2^	0.8072	0.9901	0.9217
		PSO	*k*_2_ (g mg·min^−1^)	0.58	0.06	0.04
			*q_e, calc_* (mg g^−1^)	0.93	4.90	9.01
			R^2^	0.9423	0.9875	0.9774
			*q_e, exp_*	0.94	4.72	8.93

## Data Availability

The relevant data from this research are available in the authors’ repositories.
